# Long Non-Coding RNAs Dysregulation and Function in Glioblastoma Stem Cells

**DOI:** 10.3390/ncrna1010069

**Published:** 2015-06-03

**Authors:** Xiaoqin Zhang, Karrie Meiyee Kiang, Grace Pingde Zhang, Gilberto Kakit Leung

**Affiliations:** Department of Surgery, Li Ka Shing Faculty of Medicine, The University of Hong Kong, Hong Kong, China; E-Mails: zhxq@connect.hku.hk (X.Z.); kkarrie@connect.hku.hk (K.M.K.); pingder@hku.hk (G.P.Z.)

**Keywords:** glioblastoma stem cell, lncRNA, transcription factor, miRNA, lncRNA-protein interaction, lncRNA-miRNA interaction, signal pathway

## Abstract

Glioblastoma multiforme (GBM), the most common form of primary brain tumor, is highly resistant to current treatment paradigms and has a high rate of recurrence. Recent advances in the field of tumor-initiating cells suggest that glioblastoma stem cells (GSCs) may be responsible for GBM’s rapid progression, treatment resistance, tumor recurrence and ultimately poor clinical prognosis. Understanding the biologically significant pathways that mediate GSC-specific characteristics offers promises in the development of novel biomarkers and therapeutics. Long non-coding RNAs (lncRNAs) have been increasingly implicated in the regulation of cancer cell biological behavior through various mechanisms. Initial studies strongly suggested that lncRNA expressions are highly dysregulated in GSCs and may play important roles in determining malignant phenotypes in GBM. Here, we review available evidence on aberrantly expressed lncRNAs identified by high throughput microarray profiling studies in GSCs. We also explore the potential functional pathways by analyzing their interactive proteins and miRNAs, with a view to shed lights on how this novel class of molecular candidates may mediate GSC maintenance and differentiation.

## 1. Introduction

An important progress in cancer biology has been the identification of a key subpopulation of tumor cells with stem cell properties, now commonly referred to as cancer stem cells (CSCs) [1,2]. The latter make up only a small fraction of the tumor cell mass, but are thought to be able to self-renew and re-generate the parent tumor [1,2,3,4]. Glioblastoma multiforme (GBM), the most common and deadly malignant primary brain tumor, was among the first solid tumors in which the existence of CSCs was experimentally demonstrated about a decade ago [5,6,7]. Currently, glioblastoma stem cells (GSCs) have been extensively validated in various preclinical models and characterized as the tumor-initiating cells of GBM, as well as the potential reason for the tumor’s innate radio-chemo resistance [8,9,10,11,12,13,14]. These findings provided an impetus for furthering our understanding of the cellular and molecular mechanisms of tumor maintenance and recurrence in GBM.

Long non-coding RNAs (lncRNAs), which are by definition transcripts with lengths greater than 200 nucleotides and without protein-coding function, have been proposed as key regulators of diverse biological processes including cell pluripotency and tumorigenesis [15,16,17,18]. They are aberrantly expressed in a variety of diseases, and may mechanistically interact with key proteins or RNAs to execute their biological functions at various levels [16,18,19]. Increasing evidence shows that abnormal expression of lncRNAs may alter basic cellular biological processes and contribute to the malignant phenotypes in GBM [20,21,22,23,24]. Moreover, differential expression of specific lncRNAs may also correlate with the tumorigenic properties of GSCs [25].

In this review, we summarize currently available evidence regarding the potential associations between lncRNAs and GSCs. First, we review the current knowledge about the definitions, characteristics and biomarkers of GSCs. Second, we provide a comprehensive summary of known lncRNA dysregulations in GSCs by screening existing microarray gene expression data. Finally, we discuss the potential functions and mechanisms of these lncRNAs in GSC maintenance and differentiation by analyzing the formers’ interactive transcription factors (TFs), miRNAs as well as RNA binding proteins (RBPs).

## 2. GSCs-Definitions, Characteristics and Biomarkers

**Figure 1:**
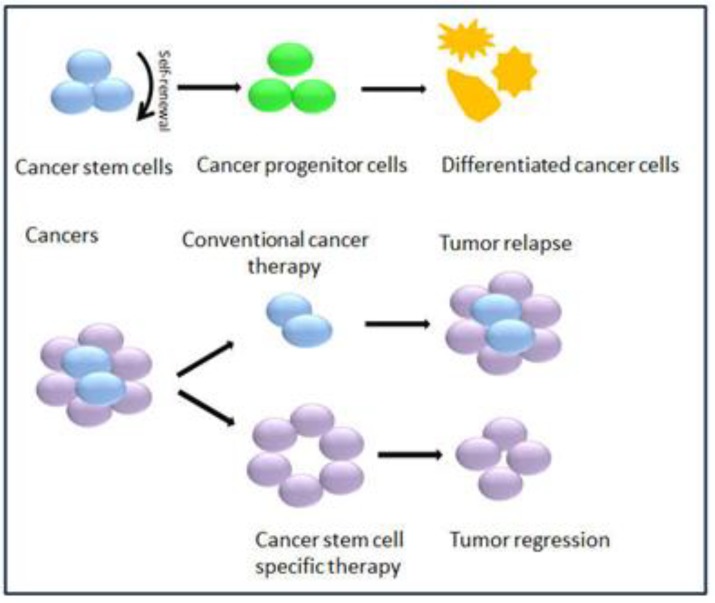
Cancer stem cell theory.

Despite continued efforts, there is as yet no consensus within the scientific community on how to define CSCs and thus GSCs. According to the American Association of Cancer Research (AACR) workshop, CSCs are a subpopulation of cells that have the capacities for self-renewal and differentiation into heterogeneous subpopulations of cancer cells that comprise the tumor [26]. To be identified as GSCs, therefore, glioblastoma cells must possess the ability of sustainable neurosphere formation in culture and tumor generation *in vivo* (self-renewal), as well as the ability to differentiate into multiple neural lineages that recapitulate the initiate tumor pathology (multiple differentiation) [27,28]. From a clinical point of view, GSCs are thought of as the small fraction of malignant cells that may survive conventional chemo-and radiotherapy, and regenerate recurrent tumors [12,29,30] (Figure 1).

While there has been considerable interest in studying CSCs derived from GBM tissues, isolating this sparse population of cells with high yield and viability from tumor bulks has been a challenge. These cells have been isolated in serum-lacking media containing growth factors, and would aggregate into spheres in suspension [5,6,7]. The main advantage of the serum-lacking culture method is the greater preservation of native phenotypes and genotypes, which are less well preserved in cells cultured in serum-containing medium because of the accumulation of aberrations over repeated passaging [31]. In contrast to the hyper-proliferative and hyper-angiogenic phenotypes of glioblastoma tumors, GSCs possess neuroectodermal properties, and express genes associated with neural stem cells, radial glial cells and neural crest cells, while portraying also a migratory, quiescent and undifferentiated phenotype [32]. Thus, cell-cycle-targeted radio-chemotherapy, which aims to kill fast-growing tumor cells, would not completely eliminate GBM tumors [32].

GSCs have biomarkers that are distinct from those found in differentiated tumor cells, and the isolation of GSCs based on surface markers is now feasible [33]. Currently, a subset of identifiable surface markers, summarized in several comprehensive reviews [14,28,34,35], has been used to isolate and characterize GSCs even though their specificities remain controversial. These include CD133, A2B5, Musashi-1 and Nestin. Amongst these, CD133, also referred to prominin-1, is the most widely studied and employed marker for GSCs. CD133 is a trans-membrane protein with no clearly-defined function [36,37]. Multiple independent studies found that CD133 + GBM cells fulfilled the definition of GSCs in that they had higher colony formation efficiency, multilineage differentiation capacity, and an increased ability to form tumors in serially transplantable xenografts when compared to CD133 − GBM cells [5,6,30,32,38,39]. However, subsequent studies revealed that CD133 did not consistently distinguish GSCs from non-GSCs, and CD133− cells may also be tumorigenic when xenografted *in vivo* [40,41]. These discrepancies illustrate the potential heterogeneity of GSCs as well as the necessity to employ combined markers or methods (such as functional identification) in enriching and validating GSC populations.

## 3. LncRNAs Dysregulated in GSCs

The distinct growth behaviors and characteristics of GSCs mean that it is intellectually attractive and clinically relevant to identify the underlying molecular characteristics, which may provide new insights into the elimination of this subpopulation of cells and provide curative strategies. Previous genome-wide profiling studies have identified aberrantly expressed protein-coding genes and miRNA genes associated with GSCs [39,40,41,42]. In contrast, dysregulation of lncRNAs in GSCs at individual gene levels have not been reported until recently; the global and comprehensive lncRNA transcriptome features in GSCs remain largely unknown. Our previous studies and several other independent studies showed that lncRNA profiling could actually be achieved by mining the existing microarray gene data, such as the Affymetrix microarray datasets [43,44,45,46]. Based on this approach, the dysregulated lncRNAs associated with GSC properties are comprehensively screened and summarized in this present review. The published GSC microarray gene expression study (on Affymetrix HG U133 Plus 2.0 platform) used for lncRNA mining here, as well as the dysregulated lncRNAs identified from them, are summarized in Table 1.

**Table 1:** Published GSC profiling studies as well as the dysregulated lncRNAs identified.

Authors ^1^	Year	Samples	No. of	No. of	Ref.
			Up-regulated LncRNAs (≥2.0 fold) ^2^	Down-regulated LncRNAs (≥2.0 fold) ^2^	
**Dysregulated LncRNAs between GSCs and Differentiated GBM Cells Comparison**					
Araki *et al.*	2013	GSC (sphere) *vs.* differentiated GBM cells	6: LOC100127888, H19, RP11-112J3.16, *et al.*	28: DLX6-AS, LOC643763, FLJ39609, *et al.*	[47]
Aldaz *et al.*	2013	GSC (sphere) *vs.* differentiated GBM cells	28: H19, MIAT, LOC150622, LOC100127888, XIST, RP11-112J3.16, *et al.*	11: RP11-346D6.6, C6orf155, HCG4, FLJ39609, *et al.*	[42]
**Dysregulated LncRNAs between GSCs with Different Subtypes**					
Beier *et al.*	2007	CD133 + GSCs *vs.* CD133 − GSCs	38: XIST, H19, HOTAIR, LOC100192378, AC006213.1, MIAT, *et al.*	34: CTC-231O11.1, RP11-745C15.2, LOC100130776, C14orf139, *et al.*	[40]
Gunther *et al.*	2008	CD133+ GSCs *vs.* CD133 − GSCs	51: H19, RP11-331K15.1, RP11-547I7.2, LOC100192378, MIAT, HOTAIR, *et al.*	10: C14orf139, DLX6-AS, MIR155HG, LOC100130776, *et al.*	[41]
**Dysregulated LncRNAs between GSCs and NSCs Comparison**					
Rheinbay, *et al.*	2013	GSCs *vs.* NSCs	173: LOC399959, LOC645323, HOTAIRM1, H19, MALAT1, SOX2ot, *et al.*	19: HYMAI, AL133167.1, FLJ31485, *et al.*	[58]

^1^ Profiling studies searching was performed in public GEO database (December, 2014). Only the datasets profiled on Affymetrix HG-U133 Plus 2.0 microarray platform were enrolled in our review analysis. With regard to how to process Affymetrix HG-U133 Plus 2.0 raw data and mine lncRNA information from it, please refer to our previous paper for details [43]. ^2^ For each individual study reviewed here, the total number of dysregulated lncRNAs, as well as the representative candidates were listed. Representative candidates were defined if they fulfilled one of following the criteria: (1) They were the top 3 dysregulated genes in comparison; (2) They appeared in more than one independent study reviewed at the same dysregulation pattern; (3) They have been functionally reported in public studies, especially in cancer.

### 3.1. Dysregulated LncRNAs during GSC Differentiation

Comparative analyses of lncRNA expression profiles in GSCs (defined by sphere formation) and their differentiated tumor cell counterparts (induced by adding serum) revealed significant differential lncRNAs dysregulations, indicating the potential roles of lncRNAs in regulating GSCs maintenance and differentiation. For example, Araki *et al.* examined the lncRNA expression profiles between GSCs derived from four different GBM patient samples and the corresponding differentiated tumor cells, and identified a set of 34 differentially expressed lncRNA transcripts (out of 2448, fold change ≥2.0) [47]. Amongst these, the most notable candidate is H19, which was one of most up-regulated lncRNAs in GSCs as compared to the differentiated cells, suggesting that H19 may have a potential role in stemness maintenance in GSCs. In support of this hypothesis, H19 has been reported as a crucial factor for the maintenance of adult haematopoietic stem cells [48].

A subsequent study that compared the gene expression patterns between GSCs and differentiated cells also observed dramatic lncRNA dysregulations. Aldaz *et al.* performed lncRNA profiling in four patient-derived GSCs (also defined by sphere formation) and the corresponding differentiated tumor cells. This revealed differential expressions of 39 lncRNAs (fold change ≥2.0) [42]. The study confirmed the up-regulation of H19 in GSCs, and also identified a large population of novel dysregulated lncRNA candidates. The most striking dysregulations were observed for MIAT, XIST, RP11-346D6.6, C6orf155 and HCG4. Amongst these, MIAT and XIST were found to be up-regulated in GSCs when compared to differentiated cells; RP11-346D6.6, C6orf155 and HCG4 were down-regulated. Consistent with these findings, the up-regulation of XIST was confirmed in another study, in which XIST was found to have higher expression in GSCs and may regulate the GSCs growth both *in vitro* and *in vivo* [25].

However, it is important to note that there was little overlap between the lists of dysregulated lncRNAs from the above two studies; the degree of between-study concordance was low. For example, amongst the 34 lncRNAs identified in Araki’s study [47] and the 39 in Aldaz’s study [42], only four lncRNAs were identified in both studies, including the above mentioned H19. The similar situation was previously reported in miRNA profiling studies in glioma also [49]. The large variability in patient samples, as well as discrepancies in the choice of GSCs-maintaining medium may underlie this disparity.

### 3.2. Dysregulated LncRNAs between GSCs with Different Subtypes

LncRNAs are also differentially expressed in different subtypes of GSCs. It has been reported that GSCs may have different sub-phenotypes and thus growth properties [40,41]. For example, GSCs with positive CD133 expression showed a spherical growth pattern (non-adherent) *in vitro* and would form highly invasive tumors *in vivo*, while GSCs with negative CD133 expression demonstrated a semi-adherent (or adherent growth) in culture and reduced tumor invasion in animals [40,41]. Comparative analysis of lncRNA profiles between these two subtypes of GSCs revealed significantly dysregulated lncRNAs, which may provide clues for the molecular mechanisms that underlie the differences in their growth phenotypes. For example, by comparing the lncRNA profiles of three CD133 + GSCs and three CD133 − GSCs, Beier *et al.* identified a set of 72 differentially expressed lncRNA transcripts (fold change ≥2.0) [40]. Among these, the expression levels of XIST, H19, and HOTAIR were markedly higher in the CD133 + GSCs than in the CD133− ones; while the expression levels of CTC-231O11.1, RP11-745C15.2, LOC100130776, and C14orf139 were significantly lower in the CD133 + GSCs than in the CD133− ones (Table 1). Of these, the up-regulations of H19 and HOTAIR were confirmed by an independent subsequent study [41]. In support of this hypothesis, H19 and HOTAIR have been reported to increase the propensities for tumor metastasis in bladder cancer and breast cancer [50,51,52].

### 3.3. Dysregulated LncRNAs between GSCs and Neural Stem Cells (NSCs)

GSCs have similar but not identical characteristics to that in non-malignant NSCs [53,54,55,56,57]. The two cell types share some surface markers, can both divide and give rise to daughter stem cells with capabilities identical to that of the parental cells (self-renewal), and can both differentiate into multiple neural cell types (multipotency) [53,54,55,56,57]. However, unlike NSCs, GSCs act in a dysregulated manner and possess tumorigenic characters when implanted into immune-deficient animals [55]. By comparative analysis of lncRNA expression profiles between GSCs and non-malignant NSCs, Rheinbay *et al.* revealed the significantly differential expression for HOTAIRM1, H19, MALAT1 and SOX2ot [58]*.* The dramatic up-regulations of their expressions in GSCs indicate their potential oncogenic roles in the malignant transformation of NSCs. In agreement with this hypothesis, H19, MALAT1 and SOX2ot have been reported to be tumorigenic or to function as metastasis promoters in multiple cancer types [50,59,60,61,62].

## 4. Functional Roles and Molecular Mechanism of LncRNAs in GSCs

While the significant lncRNA dysregulations observed above would suggest their potential roles in GSCs, the precise functions and molecular mechanism by which these lncRNAs may operate remain incompletely understood. There is currently little evidence to directly link these lncRNAs with specific cellular processes or signaling pathways in GSCs. However, it has been generally accepted that lncRNAs may function through interactions with their molecular partners, such as proteins and RNAs [19,63,64]. Therefore, analyzing the binding potentials of lncRNAs with their interactive molecules may theoretically help predict the formers’ functions and mechanisms.

**Figure 2:**
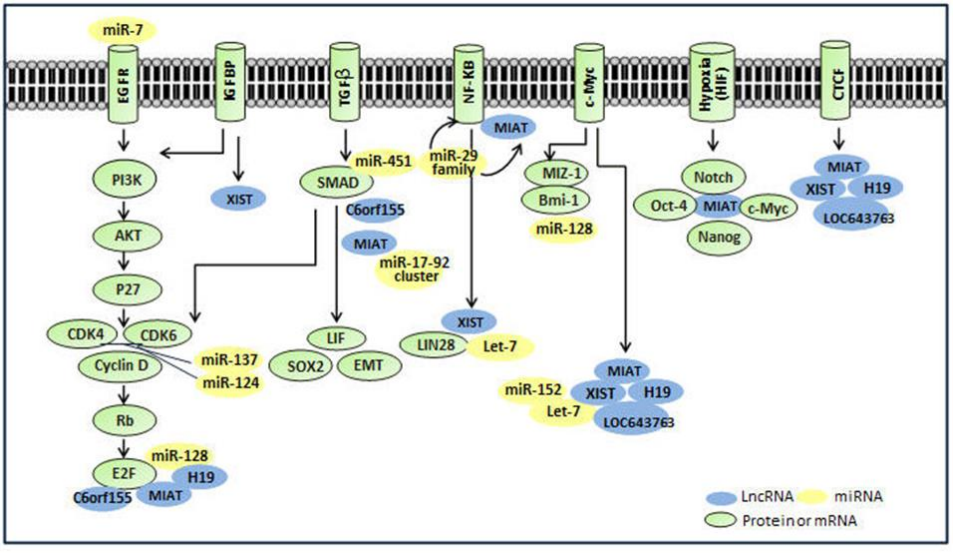
Representative figures of lncRNA interaction with key molecules or cellular processes in GSCs.

To decipher the functional roles of lncRNAs in GSCs, we have examined extensively the interactive potentials of various identified lncRNAs with transcription factors (TFs), RNA binding proteins (RBPs) and miRNAs, by using currently available research tools [65,66]. It is indeed interesting to find that lncRNAs may show strong binding potentials with some key transcription factors, miRNAs or gene pathways that are also crucial for the “stemness” maintenance in GSCs. A discussion of these findings from bioinformatics analyses by means of publicly available tools [65,66], and with a relevant literature review, is provided in the following sections and in Figure 2. Related interactive transcription factors, miRNAs and RNA binding proteins are summarized in Table 2.

### 4.1. Interaction with Stem Cell Transcription Factors (TFs)

Several transcription factors, such as c-Myc, OCT-4 and Nanog, have been shown to play key roles in promoting and stabilizing the “stem-cell-like” phenotype of non-malignant stem cells and GSCs [67,68,69,70,71,72,73,74]. These factors may activate the expression of a large number of downstream genes, enhance self-renewal, and inhibit the differentiation of stem cells through various pathways [67,68,69,70,71,72,73,74]. The lncRNA-TF interaction analysis, based on the ChIP-Seq data, revealed numerous binding sites for key transcription factors in the promoter areas (5 kb upstream and 1 kb downstream) of these lncRNAs, implying that lncRNAs may act as mediators of these key transcription factors in regulating GSCs maintenance.

**Table 2:** Interaction of lncRNAs with TFs, miRNAs and RBPs ^1^.

LncRNAs ^2^	Interactive TFs ^3^ (Number of Binding Sites)	Interactive miRNAs ^3^	Interactive RBPs ^3^
H19	NFKB (39), E2F (30), c-Myc (45), CTCF (60)	miR-29a, miR-29b, miR-29c, miR-18a*,* miR-19a, miR-20a, miR-19b, *et al.*	NA
MIAT	NFKB (45), E2F (43), Nanog (27), SMAD (20), c-Myc (15), Oct-4 (5), CTCF (20)	miR-29a, miR-29b, miR-29c, and miR-150	NA
XIST	NFKB (18), TAF1 (14), c-Myc (8), CTCF (8), Nanog (2), HNF4A(6)	miR-124, miR-34a, miR-137, miR-146a, miR-326, miR-7 and miR-425, miR-152, let-7, *et al.*	LIN28, IGF2BP
LOC100127888	NA	NA	NA
RP11-112J3.16	HNF4A (1)	NA	NA
LOC643763	CTCF (3), c-Myc (1), NFKB (1), Nanog (1)	NA	NA
RP11-346D6.6	CDX2(2), GATA6 (3), HNF4A (3), Nanog (1)	NA	NA
FLJ39609	CDX2(2), c-Myc (1), USF-1 (4)	NA	NA
C6orf155	CDX2(5), E2F (10), HNF4A (4), Nanog (5), SMAD (5)	NA	NA
HCG4	NA	NA	NA
DLX6-AS	NA	NA	NA

Abbreviations: TF, transcription factor; RBP, RNA binding protein; NA, not applicable. ^1^ The interactions of lncRNAs with miRNAs and RBPs were analyzed by using the public tool starbase v2.0 [Ref.65], and the interactions with TFs were analyzed by using the public tool ChIPBase [66]. Due to space limit. ^2^ Only lncRNAs indicated for GSCs maintenance (dysregulated in GSCs and differentiated GBM cells comparison) were enrolled for analysis here. ^3^ Only molecules that have been functionally reported in stemness regulation were included in the table.

An example is MIAT, which is one of the most highly upregulated lncRNAs in GSCs as compared to the differentiated GBM counterparts in Aldaz’s study [42]. By screening its promoter area, enriched binding sites were found for all the above three TFs: 27 binding sites for Nanog, 15 for c-Myc and 5 for OCT-4 (Table 2). While the functional role of MIAT in GSCs is not known, MIAT has been reported to interact with OCT-4 and may play regulatory roles in mouse embryonic stem cells [75]. Moreover, MIAT has been found to be dysregulated during the differentiation of normal neural stem cells [76]. Its strong interaction with multiple key stem-cell-associated transcription factors and its significant dysregulation in GSCs suggest that MIAT may play important roles in regulating GSCs, and warrants further studies.

Another interesting example is H19, which was one of the most up-regulated lncRNAs in GSCs as compared to differentiated GBM cells in the two aforementioned studies [42,47]. H19 was found to possess 45 c-Myc binding sites in its promoter area. As the first reported lncRNA in mammalian cells, H19 has been extensively studied in developmental biology as well as oncology during the past two decades [77,78]. Supporting our observation here, Barsyte *et al.* have shown that c-Myc could significantly induce the expression of H19 in T98G GBM cells through direct binding [79]. Although the tentative link between H19 and GSCs is yet to be confirmed, H19 has been found to regulate stemness in haematopoietic as well as embryonic stem cells [48,80]. How H19 may interact with c-Myc and regulate GSCs growth properties deserves further investigations.

A significant lncRNA-TF interaction was also observed for another stem-cell-associated transcription factor, CTCT. CTCF (CCCTC-binding factor) is a highly conserved multifunctional DNA-binding protein with thousands of binding sites at the genome-wide level [81]. It can act as a transcriptional activator, repressor and insulator. It can also attract many other transcription factors, transcription activators or repressors to chromatin, and can thus play extensive regulatory roles in gene expressions [81,82]. CTCF is associated with some biological processes, including embryonic stem cell differentiation [83], neuronal [84] and haematopoietic development [85,86]. Here, we found that multiple lncRNAs, including H19, MIAT, XIST and LOC643763, contained abundant binding sites with CTCF (Table 2), suggesting the potential roles of CTCF in maintaining GSCs. Of even greater importance are XIST and H19, which contain 20 and 60 CTCF binding sites, respectively. It is therefore an attractive idea to investigate whether and how these lncRNAs would interact with CTCF and play regulatory roles in GSCs. In supporting our observation here, CTCF has actually been reported to mediate the imprinted expression of H19, as well as its neighbor gene IGF2 by means of methylation-dependent binding [87].

### 4.2. Interaction with MiRNAs

GSCs-associated lncRNAs also contained enriched binding sites with miRNAs that have been reported to be functional in GSCs, suggesting that interaction with miRNA may be another potential functional entity of lncRNAs in GSCs. It has been suggested that lncRNAs and miRNAs might participate in a shared competing endogenous RNA (ceRNA) regulatory network, since they may actually regulate each other reciprocally [88,89]. In this ceRNA network, miRNA can regulate lncRNAs as they do on mRNAs since lncRNAs also have similar miRNA targeting sites as mRNAs as shown in a recent global analysis of Argonaute (Ago)-bound transcripts using the HITS-CLIP technique [65,90]. At the same time, lncRNA can reversely regulate miRNAs through their abilities to compete for miRNA binding, and to act as miRNA sponge or host gene [91,92,93].

A notable example of lncRNA-miRNA interaction is XIST. This lncRNA was found to possess binding sites with almost all the well-characterized GSCs-associated miRNAs, suggestive of its potential role as a super “miRNA sponge”. These include miR-124, 34a, miR-137, miR-146a, miR-326, miR-7 and miR-425 (Table 2). All these miRNAs are down-regulated in GSCs, and may regulate GSCs growth behavior by targeting different downstream targets [35,94]. Additionally, XIST also demonstrates binding potentials with other multiple miRNAs, which have previously not been functionally identified in GSCs. These include miR-152 and let-7 family members. This is in agreement with a recent study, which demonstrated a reciprocal regulation between XIST and miR-152 in GSCs [25]. In this interesting study, the authors first determined that XIST was up-regulated in GSCs, and that the knock-down of XIST would suppress GSCs growth *in vitro* and tumorigenicity *in vivo*. Further analyses revealed that there was reciprocal repression between XIST and miR-152: knock-down of XIST may up-regulate miR-152, and *vice versa* [25]. The study provided the earliest evidence of lncRNA-miRNA interactions in GSCs. As for the let-7 family, it has been reported to regulate tumorigenicity in breast cancer stem cells [95]. The enriched binding targets existed for almost all the let-7 family members in XIST (let-7a, b, c, d, e, f, g). Together with other evidence (detailed in 4.3 below), these findings suggested that XIST may be another potential target in GSCs regulating.

Significant lncRNA-miRNA interactions have also been observed for MIAT and H19 (Table 2). Both may act as the targets for miR-29 family (a, b, c), which has been widely reported to regulate cell proliferation, migration, invasion and tumorigenesis in GBM [42,96,97]. Additionally, H19 was found to possess targeting sites for multiple members of the miR-17-92 cluster. The latter has previously been implicated in the regulation of GBM neurosphere formation (presumably stem cells), differentiation, apoptosis and proliferation [98]. Inhibition of miR-17-92 reduced apoptosis and decreased cell proliferation in GBM neurospheres. These findings therefore indicated that H19-miR-17-92 cluster interaction may be one of possible ways in mediating the GSCs maintenance and differentiation.

### 4.3. Interaction with NFKB Pathways

The presence of extensive direct binding sites for NFKB or other key member genes in NFKB suggest the potential involvement of lncRNAs in this important signaling pathway. NFKB is a transcription factor and an inducer of signal pathway in glioma [99]. NFKB has been reported to regulate GSCs maintenance independently or in conjunction with the STAT and Notch pathways [100,101]. It was found that MIAT has 45 binding sites with NFKB in its promoter area, suggesting the involvement of NFKB and MIAT in each other’s signaling pathway. Another piece of supporting evidence for there being a connection between MIAT with NFKB pathway is their interactions with miR-29. As mentioned above, MIAT has enriched target sites for miR-29. It is interesting to found that miR-29 is also an important mediator of NFKB. NFKB could suppress miR-29 transcription and promoter function. It is thus tempting to speculate whether NFKB, miR-29 and MIAT may interact.

Another lncRNA that showed interactions with NFKB is, again, XIST. The direct evidence is that XIST possess 18 NFKB binding sites in its promoter area. The indirect evidence is that XIST may interact with LIN28, a key member of NFKB pathway [102]. LIN28 is a conserved RNA-binding protein (RBP) implicated for pluripotency, reprogramming, and oncogenesis [103,104,105,106]. It was previously shown that Lin28 expression could be activated directly by NFKB [102]. At the same time, LIN28 was able to decrease let-7 miRNA levels [102,107,108,109], and as well as the other downstream effects, such as the activation of STAT3 transcription factor [110]. This NFKB-LIN28-let-7 axis has been reported to be able to transform immortalized breast cells into self-renewing mammospheres that contain CSCs [102]. The interactions of XIST with NFKB, LIN28 and let-7 family mentioned above indicate the possible roles of XIST in maintaining GSCs properties through this axis. The detailed interactive mechanisms of XIST in this axis, however, need to be further studied.

### 4.4. Interaction with Other Molecules or Pathways

LncRNAs also has the tendency to interact with other well-characterized molecules or important cellular processes in GSCs. A detailed description is beyond the scope of this review. To cite an example, MIAT has been shown to be involved in TGF-beta signaling, since the former has 20 binding sites for the key signal transducer gene of the latter pathway-SMAD [111,112,113]. Three lncRNAs, including RP11-346D6.6, FLJ39609 and C6orf155, possess enriched binding sites with CDX2, indicating the potential interactions of lncRNAs in CDX2-mediated cellular processes crucial for the pluripotency maintenance [114]. Another three lncRNAs, C6orf155, MIAT and H19, also showed strong interactions with the cell-cycle gene E2F, an important family of transcription factors that regulate cell cycle progression and thus cell proliferation [115,116,117].

## 5. Conclusions

Growing evidence has shown that GSCs, which possess resistance to radiation therapy and chemotherapy, are responsible for tumor initiation and propagation in GBM. These characteristics indicate that GSCs are promising therapeutic targets, and that eliminating GSCs may improve patient outcome. Successful targeted therapies depend heavily on the identification of unique markers and signaling pathways in GSCs that can distinguish them from both normal and the non-GSC tumor cells. The functional significance of lncRNAs in GBM and other cancer types is beginning to emerge. The identification of lncRNAs that are dysregulated in GSCs, as well as their potential functional mechanisms, will present researchers with many opportunities for the studying of GBM initiation and progression as well as the development of future treatment for glioma. Further researches are needed to identify and verify the functions of different lncRNAs in not only GBM but also other cancers.
